# Chromatin accessibility reveals potential prognostic value of the peak set associated with smoking history in patients with lung adenocarcinoma

**DOI:** 10.1016/j.heliyon.2024.e41006

**Published:** 2024-12-06

**Authors:** Han Liang, Jianlian Deng, Tian Luo, Huijuan Luo, Fuqiang Li, Kui Wu, Cong Lin

**Affiliations:** a HIM-BGI Omics Center, Hangzhou Institute of Medicine (HIM), Chinese Academy of Sciences (CAS), BGI Research, Hangzhou 310000, China; b Guangdong Provincial Key Laboratory of Human Disease Genomics, BGI Research, Shenzhen 518083, China; c BGI Genomics, Shenzhen 518083, China; d College of Life Sciences, University of Chinese Academy of Sciences, Beijing 100049, China

**Keywords:** ATAC-Seq, Network, LUAD, Smoking, Prognostic

## Abstract

Considerable differences in molecular characteristics have been defined between non-smoker and smokers in patients with lung adenocarcinoma (LUAD), yet studies on open chromatin patterns associated with LUAD progression caused by smoking are still lacking. Here, we constructed a novel network based on correlations between each ATAC-seq peak from TCGA data using our previously developed algorithm. Subsequently, principal component analysis was performed on LUAD samples with retained peaks filtered by the correlation network, and pathway analysis was conducted to identify potential pathways involved. We identified a set of peaks that discriminated smokers in LUAD patients according to levels of exposure to tobacco quantified in pack-years. These peaks were also significantly associated with progression-free survival and overall survival of these patients. Further examination of the gene set related to those peaks revealed that the comprising genes, such as *KRT19*, *B3GNT3, CLDN7* and *CLDN3* are strongly associated with LUAD development. They are consistent with the important roles of the associated pathways in LUAD oncogenesis induced by smoking, including estrogen response, apical junction and glycolysis pathways. In summary, our study may provide valuable insights into exploring ATAC-seq peaks and understanding smoking-related LUAD carcinogenesis from a perspective of open chromatin changes.

## 1 Introduction

Lung cancer remains the leading cause of cancer death with over 1.8 million deaths annually, and the incidence of lung cancer is still increasing worldwide [1,2]. More than 85 % of lung cancer cases are diagnosed as non-small-cell lung cancer (NSCLC), with lung adenocarcinoma (LUAD) and lung squamous cell carcinoma (LUSC) being the two main histological subtypes. LUAD alone accounts for approximately 40 % of NSCLC cases, resulting in over 500,000 deaths per year globally [2,3]. The most important risk factor for lung cancer is still cigarette smoking, which is responsible for about 85–90 % of all cases [2,4]. Pack-years is the standard measure used to quantify the amount of cigarette smoking over a person's lifetime, with one pack-year corresponding to smoking a pack of cigarettes daily for one year. A cumulative exposure to tobacco of 10–20 pack-years is reported to be associated with a clinically relevant increase in morbidity [5]. In the context of lung cancer screening, smokers with a smoking history ≥20 pack-years is one of the major criteria recommended by the National Comprehensive Cancer Network and the American Association for Thoracic Surgery [6]. The other emerging risk factors include second-hand smoking and air pollution [7], such as PM2.5 (Particulate Matter 2.5 describes particles that are 2.5 μm or smaller and harmful for human respiratory system), which is claimed to cause lung cancer in many developing countries [8,9]. Although NSCLC is strongly associated with smoking, LUAD is more common in never-smokers [2,10]. However, compelling evidence indicates that never or light -smoker patients with LUAD have a significantly better survival rate than smokers, suggesting different levels of smoke exposure may cause distinct molecular mechanisms underlying their clinical difference [[11], [12], [13]].

Recent efforts have focused on characterizing various molecular alterations in LUAD using high-throughput genome sequencing, leading to comprehensive profiling of different oncogenic driver mutations [14,15]. Besides *EGFR* mutations and *ALK* fusions, for which targeted therapies have become the standard treatment for LUAD, several other activated oncogenes such as*, KARS, TP53, ERBB2* and *BRAF* are also found in LUAD [16,17]. As in-depth multi-omics studies continue to progress, striking differences in molecular characteristics have been discovered between LUAD never-smokers and smokers. For example, LUAD patients with different levels of tobacco consumption exhibit different mutation frequencies in *EGFR, TP53* and *KRAS* genes, with *EGFR* mutations occurring more frequently in never smokers [10,18]. In addition, gene expression analysis identified distinct patterns of dysregulated genes in smokers with LUAD, with associated altered pathways are particularly involved in the cellular immune response and cell cycle regulation [19,20]. Epigenetic studies also demonstrated clear differences in methylation profiles between LUAD in never smokers and smokers [[21], [22], [23]]. To date, however, other epigenetic studies, such as those investigating open chromatin patterns associated with LUAD progression caused by smoking, are still lacking. Different from whole genome (exome) sequencing, which identifies genetic risks, the study of open chromatin regions can offer insights into epigenetic and regulatory modifications, and thus may provide novel genes or pathways that are involved.

Recently, assay for transposase accessible chromatin sequencing (ATAC-seq) has emerged as a powerful tool for profiling chromatin accessibility in different human diseases and has advanced our understanding of the coordination of gene expression processes [24,25]. Until now, only a few studies have explored open chromatin states in NSCLC with ATAC-seq. An elegant work by Corces et al. studied chromatin accessibility of 410 tumor samples from The Cancer Genome Atlas (TCGA), which included 38 cases of NSCLC [26]. More recently, an integrative analysis linking the open chromatin variations to genomic alterations among NSCLC patients has provided a comprehensive open chromatin landscape of NSCLC [27]. However, emphasis has not yet been placed on linking the clinical variables, such as cigarette smoking history to open chromatin patterns in LUAD. In this study, we first generated a network based on correlations between peaks identified from ATAC-seq data of TCGA. Using retained peaks filtered by the correlation network, we subsequently studied differences between never or light smokers (<20 pack-years) and heavy smokers (≥20 pack-years) in LUAD patients, and further identified a set of peaks and their related pathways that may associate with patients’ progression-free survival (PFS) and overall survival (OS).

## 2 Materials and methods

### 2.1 ATAC-seq data analysis

**Fig. 1:**
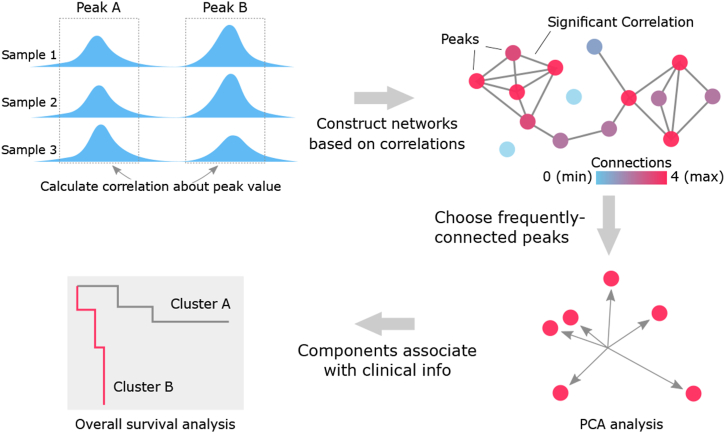
**The graphical abstract of analyses performed in this study.** To reduce the complexity of the large data and only emphasize on important peaks, a correlation network was constructed. Peaks were connected to each other if their direct or indirect correlations are significant (red dots). We chose the 10 % most frequently-connected peaks as the important peaks for the further PCA analysis. Finally, we analyzed the associations between components obtained from PCA and patient's survival.

ATAC-seq peaks were retrieved from ATAC-seq data across 23 cancer types profiled by Corces et al. (the supplementary table Data S2) [26], following the standards of ENCODE, after which high quality fixed-width peaks of 501bp were identified in samples with transcription start site (TSS) enrichment value > 5. We then used a previously described analysis method on peaks selecting and exploring [28].^2^ Briefly, we selected the available data from TCGA according to peak's quality. A peak would be considered low-quality if it has a same value in more than 5 % patients from single type of cancer, as the repeat values were likely produced by nonsense 0s (which were most likely produced by the regions that were not covered by any reads) before normalization. Eventually, we obtained 64,316 peaks across 386 samples from TCGA dataset. To further reduce the scale of data, we then applied the previously developed algorithm on the correlation network construction with retained peaks from TCGA, in which two peaks would be connected if their direct or indirect correlation is significant calculated on peak height. Detailed calculation could be found in the preprint manuscript, and code for analyses has been deposited on Github [28] (Fig. 1). We considered that the direct correlation between two peaks is significant, if the absolute value of correlation coefficient is not less than 0.4, considering the noise level is around 0.2. Furthermore, we considered that the indirect correlation between the two peaks is significant, if their direct correlations with the third peak are both significant. Here we allowed the indirect correlation to amplify peaks' aggregation effect in the network. To assess the correlation between peaks more reliably, outlier values were removed before calculating correlation [29]. Outlier peaks were calculated with the method previously described.^3^ Speciﬁcally, a value out of range [Q1-1.5 × (Q3-Q1), Q3+1.5∗(Q3-Q1)] would be considered as an outlier, where Q3 was the third quartile (75th percentile) and Q1 was the first quartile (25th percentile). In practice, the sum of all values on one axe is 0, the absolute value of each sample meant its absolute distance from the axis. We used the function “cor” from R package “stats” V3.6.2 with default arguments on the absolute values. We selected the 10 % most frequently-connected peaks for the further analysis, as those peaks were more likely to be the hub peaks in the network with high connectivity.

The unsupervised classification method principal component analysis (PCA) was used to analyze the selected ATAC-seq peaks from 22 LUAD patients in TCGA dataset. We used the function “PCA” from R package “FactoMineR” V1.34 with default arguments which produced the five most important components [30]. The association between components and smoking status were checked by the distribution of samples classified by components. To statistically assess the difference between distribution distance of samples from LUAD patients with different smoking histories (based on pack-years of smoking, status of smoking exposure was classified as light smoker (<20 pack-years) and heavy smoker (≥20 pack-years)), two-tailed unpaired t-tests were performed. Outliers were excluded based on Grubbs' test. *P* < 0.05 were considered significant.

### 2.2 Survival analyses

Cox's proportional hazards models were used to determine the prognostic impact of clinical and the set of peaks using the R packages “finalfit” and “survival” (version: v1.0.4/v3.3–1). Univariable Cox regression was performed on the selected peak set, while multivariable Cox regression was applied to the selected peak set with co-variates including tumor stage, age, gender, and T, N, M factors. The OS and PFS curves were constructed using the Kaplan-Meier method and the differences between groups were assessed by the log-rank test, using the function “survdiff” from R package “survival” [31].

### 2.3 Pathway analysis

For the pathway analysis, peaks with peak-PC correlation value greater than 0.8 were selected. Potential pathways were identified using genes involving in these peaks with the cancer hallmark gene sets by Gene Set Enrichment Analysis (GSEA). Pathways were selected with false discovery rate (FDR) q value less than 0.05.

## 3 Results

### 3.1 A correlation network built based on ATAC-seq data from TCGA

**Fig. 2:**
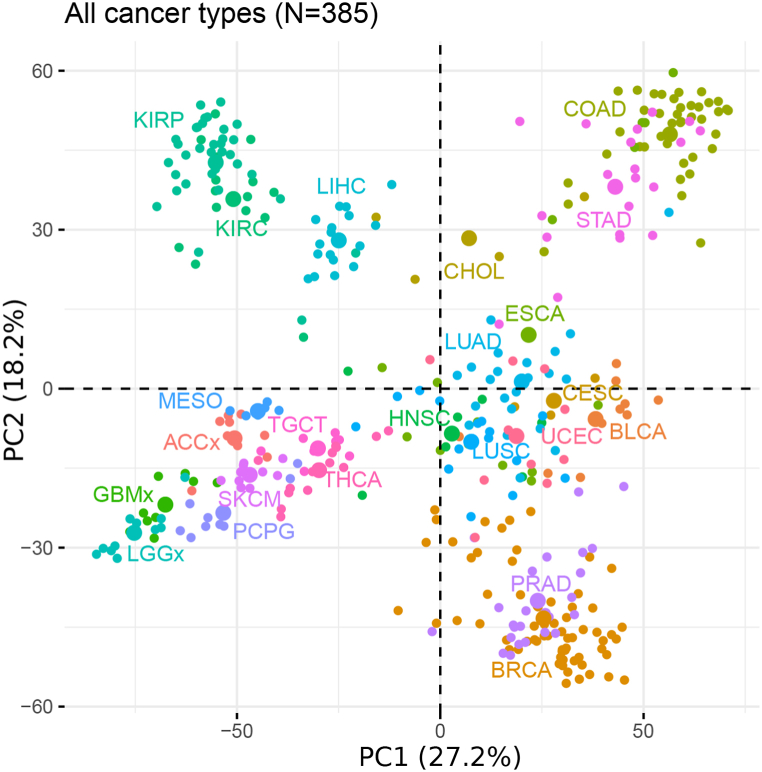
**Principal Component Analysis on the selected peaks from TCGA ATAC-data.** The unsupervised PCA for the 10 % most frequently-connected peaks selected from TCGA ATAC-seq data across all cancer types. Each dot represents a given sample. Color represents the cancer type shown in the Figure. ACC, adrenocortical carcinoma; BLCA, bladder urothelial carcinoma; BRCA, breast invasive carcinoma; CESC, cervical squamous cell carcinoma; CHOL, cholangiocarcinoma; COAD, colon adenocarcinoma; ESCA, esophageal carcinoma; GBM, glioblastoma multiforme; HNSC, head and neck squamous cell carcinoma; KIRC, kidney renal clear cell carcinoma; KIRP, kidney renal papillary cell carcinoma; LGG, low grade glioma; LIHC, liver hepatocellular carcinoma; LUAD, lung adenocarcinoma; LUSC, lung squamous cell carcinoma; MESO, mesothelioma; PCPG, pheochromocytoma and paraganglioma; PRAD, prostate adenocarcinoma; SKCM, skin cutaneous melanoma; STAD, stomach adenocarcinoma; TGCT, testicular germ cell tumors; THCA, thyroid carcinoma; UCEC, uterine corpus endometrial carcinoma.

High-quality ATAC-seq data of 410 tumor samples across 23 cancer types were downloaded and collected from TCGA. The extended peak summits with a fixed width of 501 bp were extracted from the dataset and used for further analysis [26]. We first constructed a correlation network using retained peak summits from TCGA, where two peaks were connected if their peak values were significantly correlated with each other (Fig. 1). To this end, correlations between each peak value across all samples were calculated, and the 10 % (6431) most frequently-connected peaks were chosen after removing the low-quality peaks and outliers. Using PCA on those selected peaks across all cancer types, we identified distinct clusters labeled based on different cancer-type enrichment (Fig. 2), which showed strong concordance with the t-distributed stochastic neighbor embedding (t-SNE) results from TCGA [26]. In both results, cancers originating from the same organ would group together, such as LUAD and LUSC, or kidney renal papillary cell carcinoma (KIRP) and kidney renal clear cell carcinoma (KIRC). Additionally, cancers with squamous cell types would group together, as observed in CESC, HNSC and LUSC.

### 3.2 Identification of the smoking associated peak set in LUAD patients

**Fig. 3:**
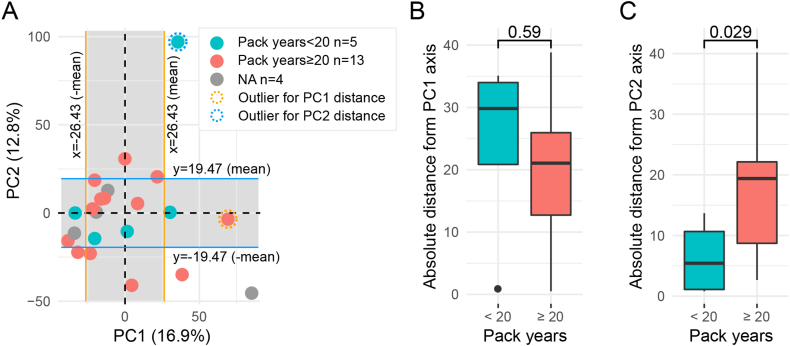
**Identification of the smoking associated peak set from LUAD patients using TCGA ATAC-data.** A. The unsupervised PCA on LUAD samples (N = 22) from TCGA. Dots present samples and their colors present patients' different smoking histories. Pack-years information of four patients is not available in TCGA data. The left and right sides of the orange borders were defined by the ±mean of all LUAD samples' distances from the PC1 axis. The upper and lower sides of the blue borders were defined by the ±mean of all LUAD samples' distances from the PC2 axis. The x and y values of each sample were generated in PCA, which were not true peaks values. The samples within the borders thus have relatively more stable PC1 or 2-related peaks. **B.** Each sample's absolute distance from PC1 axis (towards line x = 0 in Fig. 3A) was measured and compared between groups of <20 pack-years (N = 5) and ≥20 pack-years (N = 12). One outlier from group of ≥20 pack-years had been removed, according to Grubbs' test. PC1 related peak set identified from LUAD patients was not associated with smoking, as the difference is not significant. **C.** Each sample's absolute distance from PC2 axis (towards line y = 0 in Fig. 3A) was measured and compared between groups of <20 pack-years (N = 4) and ≥20 pack-years (N = 13). One outlier from group of <20 pack-years had been removed, according to Grubbs' test. *P* < 0.05 was considered statistically significant, two-tailed unpaired t-tests.

**Fig. 4:**
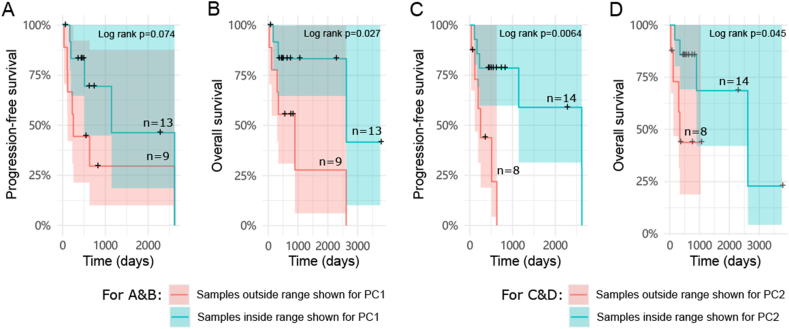
**The survival analyses of LUAD patients with different absolute distance towards PC1 or PC2.** Samples were divided into two groups based on they are inside or outside the range shown for PC1 (orange borders) in Fig. 3A. The PFS (**A**) and OS (**B**) were compared between the group of samples outside the range (N = 9) and the group of samples inside the range (N = 13) by the Kaplan-Meier survival curves. Samples were also divided into two groups based on they are inside or outside the range shown for PC2 (blue borders) in Fig. 3A. The PFS (**C**) and OS (**D**) were compared between the group of samples outside the range (N = 8) and the group of samples inside the range (N = 14) by the Kaplan-Meier survival curves. *P* < 0.05 were considered significant, log-rank test. Patients who survived but stopped being tracked are indicated by *crosses*.

We next focused on analyzing 22 LUAD patients in the TCGA dataset, using the same correlation network and methods described above. PCA results indicated that the first principal component (PC1) explained 16.9 % of the variability, while PC2 explained 12.8 % of the variability in the peaks from all LUAD samples (Fig. 3A). The LUAD samples did not form distinct patterns within the two dimensions generated by PCA analysis, interestingly however, it seemed that samples with less smoking exposure were closer to each other on the PC2 axis rather than PC1(Fig. 3A). The x or y values of each sample in Fig. 3A were generated automatically in PCA and only represented the level of variety towards PC1 or 2-axis, which are not true peaks values. Therefore, a smaller absolute x or y value of a sample meant its PC1 or 2-related peaks are less variable. Using each sample's absolute distance from the PC1 or 2-axis, we compared the difference between heavy smokers (patients with at least 20 pack-years smoking history) and the rest (Fig. 3B and C, Table S1). The results showed that the group with ≥20 pack-years indeed had a significant longer absolute distance from PC2 axis compared to the <20 pack-years group, suggesting the ATAC-seq peaks associated with PC2, rather than PC1, were influenced by smoking history. A shorter absolute distance derived from PC2 axis, representing more stable PC2-related peak values, was significantly associated with better PFS and OS of patients (Fig. 4A–D). Importantly, it was significantly correlated with better PFS independent of other clinical parameters in the multivariable Cox models, indicating a potential prognostic value for the corresponding PC2 peaks (Fig. 4C and D, Table S2). We thus further studied the gene set related to those peaks according to the defined peak-gene relationships [26] (Table S3) and explored the associated pathways. Consequently, we identified six potential pathways, including estrogen_response_late, estrogen_response_early, kras_signaling_up, apical_junction, complement and glycolysis pathways, involving 242 genes associated with the PC2-related peaks (Table 1).

**Table 1:** Six cancer-related hallmark pathways related to PC2 high contributing peaks found with GSEA.

Pathways	FDR q-value	Genes
ESTROGEN_RESPONSE_LATE	4.93E-09	*TFF3, KRT19, SFN, MAPT, PDZK1, OVOL2, TFAP2C, SERPINA1, ST14, AGR2, PKP3, EMP2, GJB3*
ESTROGEN_RESPONSE_EARLY	3.29E-08	*TFF3, KRT19, SFN, MAPT, PDZK1, OVOL2, TFAP2C, CLDN7, ELF3, AR, MUC1, LAD1*
KRAS_SIGNALING_UP	2.95E-05	*NR1H4, CCL20, USH1C, GALNT3, PLEK2, KCNN4, KIF5C, TMEM176A, HKDC1*
APICAL_JUNCTION	1.07E-02	*CLDN7, CLDN8, CDH3, PDZD3, NECTIN4, NRXN2*
COMPLEMENT	4.17E-02	*SERPINA1, HNF4A, C4BPB, KLK1, PRSS3*
GLYCOLYSIS	4.17E-02	*TFF3, ELF3, B3GNT3, CLDN3, GAL3ST1*

## 4 Discussion

During the past decade, numerous important alterations in genomes and signaling pathways caused by smoking in LUAD were unveiled by in-depth analyses. However, the precise alterations in chromatin accessibility induced by smoking remained obscure [10,19,22]. Previous research on the open chromatin landscape of NSCLC divided LUAD samples into three sub-clusters based on open chromatin peaks and identified correlations between the clusters and smoking. However, associated genes or pathways were not further studied [27]. In the present study, we first constructed a correlation network with ATAC-seq data from TCGA using the algorithm we previously developed, and analyzed 22 LUAD samples with peaks selected by the network. We determined smoking history related peaks with potential prognostic value, and subsequently found associated pathways based on the defined peak-gene relationships.

Different from the classic analyses correlating gene expression and chromatin accessibility [26,27], our study solely focused on correlations between each peak. We constructed the network based on the plausible theory that peaks highly connected with many other peaks are more likely to have a crucial function in gene regulatory processes. The correlation network assisted us in selecting critical peaks and enabled a more precise analysis for further peak classification. With this novel conception, the smoking-related peak set in this study was effectively identified from the massive ATAC-seq data. The effectiveness of this method was also demonstrated by the identification of specific mitosis-related expression patterns in the previous work on data mining of transcriptomes from 5001 cancer patients cross 22 cancer types [28]. Therefore, we believe that this method is practical for revealing crucial factors from complicated datasets and will enhance analytical capabilities for future studies of multi-omics data.

Using PCA, we identified the PC2-related peaks, which effectively differentiated heavy smokers (≥20 pack-years) from the rest of patients with LUAD tumors and associated with both PFS and OS of these LUAD patients, suggesting that genes corresponding to PC2 peaks may be influential in the carcinogenesis of LUAD caused by smoking. Indeed, pathways significantly associated with the PC2-related gene set, including estrogen_response_late/early [32,33], apical_junction [34,35] and glycolysis [36,37], are proven to play crucial roles in tumor progression.

The genes we identified through PC2-related peaks are consistent with the important role of the pathways involved in LUAD oncogenesis. Although smoking has been the leading cause of complications in NSCLC, its consequences exhibit distinct gender biases due to differences in gene and sex hormone expression [38]. In NSCLC, dysregulated pathways related to estrogen_response_late/early promote tumor proliferation, invasion and migration, potentially contributing to the gender-specific consequences induced by smoking [[39], [40], [41], [42]]. Among the identified PC2-peak related genes in those pathways, Keratin 19 (KRT19) is an intermediate filament protein that is responsible for the structural integrity of epithelial cells. Increased expression of KRT19 has been found to be correlated with tumor progression and poor prognosis in lung cancers [43]. LAD1 (Ladinin 1) in those pathways has also been found to be significantly correlated with tumor size, lymph metastasis and recurrence events, with higher expression contributing to worse survivals [44]. As for the apical_junction pathway, alterations in expression of proteins in this pathway induced by smoking have been involved in increased lung epithelial permeability and epithelial-mesenchymal transition (EMT), thereby influencing cancer progression [34,35]. Among those, claudins are important structural proteins of tight junctions located apically within the epithelial junctional complex and regulate cellular homeostasis [45]. Disrupted expression patterns in claudins can drive cell migration and invasion in lung cancers. Intriguingly, the expression of claudin 7(CLDN7), a PC2-peak related component in this pathway, has previously been found to show a significant association with smoking pack-years, with heavy-smokers showing elevated expression levels of claudin 7 [46,47]. Besides, dysregulated expressions of genes related to glycolysis pathway, for instance *B3GNT3* and *CLDN3*, have also been shown to correlate with observed malignancy in the context of LUAD progression [48,49]. Smoking can alter airway epithelial differentiation and barrier function by activating EGFR in airway basal cells, which is associated with development of smoking-associated lung cancers. In this process, *CLDN3* overexpression is modulated by the EGF pathway and has been observed to promote the malignant potential of lung adenocarcinoma [50]. Additionally, the overexpression of *B3GNT3* is specifically associated with unfavorable OS in NSCLC patients [48]. Taken together, the gene set we identified through ATAC-seq peaks comprises genes that are strongly associated with smoking status and may have potential prognostic value in patients with LUAD. However, before drawing conclusions on potential clinical implications of these genes, it is important to elucidate their functions with respect to LUAD development.

In conclusion, our study introduced a novel method to explore ATAC-seq peaks and identified a set of peaks with potential prognostic value based on chromatin accessibility alterations induced by smoking in LUAD patients. These findings provide insights into smoking-related LUAD carcinogenesis from the perspective of open chromatin alterations and may influence future clinical applications. However, further studies with larger datasets of LUAD patients are warranted to confirm the effects of PC2-related peaks exerted in this research.

## CRediT authorship contribution statement

**Han Liang:** Visualization, Methodology, Investigation, Formal analysis, Data curation. **Jianlian Deng:** Methodology, Investigation, Formal analysis, Data curation. **Tian Luo:** Visualization, Validation, Methodology, Investigation, Formal analysis. **Huijuan Luo:** Investigation. **Fuqiang Li:** Investigation, Data curation. **Kui Wu:** Supervision, Funding acquisition. **Cong Lin:** Writing – review & editing, Writing – original draft, Supervision, Methodology, Investigation, Formal analysis, Conceptualization.

## Ethics statement

Review and/or approval by an ethics committee was not needed for this study, because this study did not include human or animal participation.

## Data availability statement

The code used in current study has been deposited on Github https://github.com/HanL233/ATAC_network.

## Funding

This research was funded by the Science, Technology, and Innovation Commission of Shenzhen Municipality (grant number JCYJ20170817145454378, JCYJ20160531193931852) and the Guangdong Enterprise Key Laboratory of Human Disease Genomics (grant number 2020B1212070028).

## Declaration of competing interest

The authors declare the following financial interests/personal relationships which may be considered as potential competing interests:Kui Wu reports financial support was provided by the Science, Technology, and Innovation Commission of Shenzhen Municipality. Kui Wu reports financial support was provided by Guangdong Enterprise Key Laboratory of Human Disease Genomics.

## Footnotes

2 https://www.biorxiv.org/content/10.1101/2020.03.30.015180v1.full.

3 https://miniwebtool.com/outlier-calculator/.

## References

[1] Sung H., Ferlay J., Siegel R.L., Laversanne M., Soerjomataram I., Jemal A., Bray F. (2021). Global cancer statistics 2020: GLOBOCAN estimates of incidence and mortality worldwide for 36 cancers in 185 countries. CA A Cancer J. Clin..

[2] Herbst R.S., Morgensztern D., Boshoff C. (2018). The biology and management of non-small cell lung cancer. Nature.

[3] Herbst R.S., Heymach J.V., Lippman S.M. (2008). Lung cancer. N. Engl. J. Med..

[4] Freedman N.D., Leitzmann M.F., Hollenbeck A.R., Schatzkin A., Abnet C.C. (2008). Cigarette smoking and subsequent risk of lung cancer in men and women: analysis of a prospective cohort study. Lancet Oncol..

[5] Neumann T., Rasmussen M., Heitmann B.L., Tønnesen H. (2013). Gold standard program for heavy smokers in a real-life setting. Int J Environ Res Public Health.

[6] Boiselle P.M. (2013). Computed tomography screening for lung cancer. JAMA.

[7] Oberg M., Jaakkola M.S., Woodward A., Peruga A., Pruss-Ustun A. (2011). Worldwide burden of disease from exposure to second-hand smoke: a retrospective analysis of data from 192 countries. Lancet.

[8] Khilnani G.C., Tiwari P. (2018). Air pollution in India and related adverse respiratory health effects: past, present, and future directions. Curr. Opin. Pulm. Med..

[9] Guo H., Chang Z., Wu J., Li W. (2019). Air pollution and lung cancer incidence in China: who are faced with a greater effect?. Environ. Int..

[10] Sun S., Schiller J.H., Gazdar A.F. (2007). Lung cancer in never smokers--a different disease. Nat. Rev. Cancer.

[11] Bryant A., Cerfolio R.J. (2007). Differences in epidemiology, histology, and survival between cigarette smokers and never-smokers who develop non-small cell lung cancer. Chest.

[12] Casal-Mourino A., Valdes L., Barros-Dios J.M., Ruano-Ravina A. (2019). Lung cancer survival among never smokers. Cancer Lett..

[13] Lofling L., Karimi A., Sandin F., Bahmanyar S., Kieler H., Lambe M. (2019). Clinical characteristics and survival in non-small cell lung cancer patients by smoking history: a population-based cohort study. Acta Oncol.

[14] Weir B.A., Woo M.S., Getz G., Perner S., Ding L., Beroukhim R. (2007). Characterizing the cancer genome in lung adenocarcinoma. Nature.

[15] Cancer Genome Atlas Research Network (2014). Comprehensive molecular profiling of lung adenocarcinoma. Nature.

[16] Imielinski M., Berger A.H., Hammerman P.S., Hernandez B., Pugh T.J., Hodis E. (2012). Mapping the hallmarks of lung adenocarcinoma with massively parallel sequencing. Cell.

[17] Wu K., Zhang X., Li F., Xiao D., Hou Y., Zhu S. (2015). Frequent alterations in cytoskeleton remodelling genes in primary and metastatic lung adenocarcinomas. Nat. Commun..

[18] Le Calvez F., Mukeria A., Hunt J.D., Kelm O., Hung R.J., Taniere P. (2005). TP53 and KRAS mutation load and types in lung cancers in relation to tobacco smoke: distinct patterns in never, former, and current smokers. Cancer Res..

[19] Landi M.T., Dracheva T., Rotunno M., Figueroa J.D., Liu H., Dasgupta A. (2008). Gene expression signature of cigarette smoking and its role in lung adenocarcinoma development and survival. PLoS One.

[20] Liu Z.H., Lian B.F., Dong Q.Z., Sun H., Wei J.W., Sheng Y.Y. (2018). Whole-exome mutational and transcriptional landscapes of combined hepatocellular cholangiocarcinoma and intrahepatic cholangiocarcinoma reveal molecular diversity. Biochim. Biophys. Acta, Mol. Basis Dis..

[21] Divine K.K., Pulling L.C., Marron-Terada P.G., Liechty K.C., Kang T., Schwartz A.G. (2005). Multiplicity of abnormal promoter methylation in lung adenocarcinomas from smokers and never smokers. Int. J. Cancer.

[22] Toyooka S., Tokumo M., Shigematsu H., Matsuo K., Asano H., Tomii K. (2006). Mutational and epigenetic evidence for independent pathways for lung adenocarcinomas arising in smokers and never smokers. Cancer Res..

[23] Alexandrov L.B., Ju Y.S., Haase K., Van Loo P., Martincorena I., Nik-Zainal S. (2016). Mutational signatures associated with tobacco smoking in human cancer. Sci. Sci..

[24] Buenrostro J.D., Giresi P.G., Zaba L.C., Chang H.Y., Greenleaf W.J. (2013). Transposition of native chromatin for fast and sensitive epigenomic profiling of open chromatin, DNA-binding proteins and nucleosome position. Nat. Methods.

[25] Liu L., Leng L., Liu C., Lu C., Yuan Y., Wu L. (2019). An integrated chromatin accessibility and transcriptome landscape of human pre-implantation embryos. Nat. Commun..

[26] Corces M.R., Granja J.M., Shams S., Louie B.H., Seoane J.A., Zhou W. (2018). The chromatin accessibility landscape of primary human cancers. Science.

[27] Wang Z., Tu K., Xia L., Luo K., Luo W., Tang J. (2019). The open chromatin landscape of non-small cell lung carcinoma. Cancer Res..

[28] Liang H., Lin C., Hou Y., Li F., Wu K. (2020). Identification of the associated expression patterns as potential predictive markers for cancer prognosis. bioRxiv.

[29] Hearst M.A. (1999). Proceedings of the 37th Annual Meeting of the Association for Computational Linguistics on Computational Linguistics.

[30] Lê S., Josse J., Husson F. (2008). FactoMineR: an R package for multivariate analysis. J. Stat. Software.

[31] Harrington D.P., Fleming T.R. (1982). A class of rank test procedures for censored survival data. Biometrika.

[32] Siegfried J.M., Hershberger P.A., Stabile L.P. (2009). Estrogen receptor signaling in lung cancer. Semin. Oncol..

[33] Chakraborty S., Ganti A.K., Marr A., Batra S.K. (2010). Lung cancer in women: role of estrogens. Expert Rev Respir Med.

[34] Shaykhiev R., Otaki F., Bonsu P., Dang D.T., Teater M., Strulovici-Barel Y. (2011). Cigarette smoking reprograms apical junctional complex molecular architecture in the human airway epithelium in vivo. Cell. Mol. Life Sci..

[35] Soini Y. (2012). Tight junctions in lung cancer and lung metastasis: a review. Int. J. Clin. Exp. Pathol..

[36] Gatenby R.A., Gillies R.J. (2007). Glycolysis in cancer: a potential target for therapy. Int. J. Biochem. Cell Biol..

[37] Li X.B., Gu J.D., Zhou Q.H. (2015). Review of aerobic glycolysis and its key enzymes - new targets for lung cancer therapy. Thorac Cancer.

[38] Gazdar A.F., Thun M.J. (2007). Lung cancer, smoke exposure, and sex. J. Clin. Oncol..

[39] Couraud S., Zalcman G., Milleron B., Morin F., Souquet P.J. (2012). Lung cancer in never smokers--a review. Eur. J. Cancer.

[40] Smida T., Bruno T.C., Stabile L.P. (2020). Influence of estrogen on the NSCLC microenvironment: a comprehensive picture and clinical implications. Front. Oncol..

[41] Davuluri S., Bajpai A.K., Thirumurugan K., Acharya K.K. (2021). The molecular basis of gender disparities in smoking lung cancer patients. Life Sci..

[42] Mukherjee T.K., Malik P., Hoidal J.R. (2021). The emerging role of estrogen related receptorα in complications of non-small cell lung cancers. Oncol. Lett..

[43] Yuan X., Yi M., Dong B., Chu Q., Wu K. (2021). Prognostic significance of KRT19 in lung squamous cancer. J. Cancer.

[44] Wang Y. (2021). circ-ANXA7 facilitates lung adenocarcinoma progression via miR-331/LAD1 axis. Cancer Cell Int..

[45] Bhat A.A., Uppada S., Achkar I.W., Hashem S., Yadav S.K., Shanmugakonar M. (2019). Tight junction proteins and signaling pathways in cancer and inflammation: a functional crosstalk. Front. Physiol..

[46] Merikallio H., Kaarteenaho R., Pääkkö P., Lehtonen S., Hirvikoski P., Mäkitaro R. (2011). Impact of smoking on the expression of claudins in lung carcinoma. Eur. J. Cancer.

[47] Soini Y. (2011). Claudins in lung diseases. Respir. Res..

[48] Gao L., Zhang H., Zhang B., Zhu J., Chen C., Liu W. (2018). B3GNT3 overexpression is associated with unfavourable survival in non-small cell lung cancer. J. Clin. Pathol..

[49] Sun Y., Liu T., Xian L., Liu W., Liu J., Zhou H. (2020). B3GNT3, a direct target of miR-149-5p, promotes lung cancer development and indicates poor prognosis of lung cancer. Cancer Manag. Res..

[50] Shaykhiev R., Zuo W.L., Chao I., Fukui T., Witover B., Brekman A. (2013). EGF shifts human airway basal cell fate toward a smoking-associated airway epithelial phenotype. Proc Natl Acad Sci U S A.

